# Comparing long‐term outcomes of children treated with new‐onset type 2 diabetes in an outpatient versus inpatient setting: A retrospective chart review

**DOI:** 10.1111/1753-0407.13571

**Published:** 2024-05-16

**Authors:** Adesh Ranganna, Wenya Chen, Sean DeLacey, Juan Lado, Laura Levin, Anita Swamy, Monica E. Bianco

**Affiliations:** ^1^ Northwestern University Feinberg School of Medicine Chicago Illinois USA; ^2^ Ann & Robert H. Lurie Children's Hospital of Chicago Chicago Illinois USA; ^3^ Department of Pediatrics Northwestern University Feinberg School of Medicine Chicago Illinois USA

**Keywords:** glycemic control, insulin, long‐term outcomes, pediatric diabetes, type 2 diabetes mellitus

## Abstract

**Background:**

Early identification and management of pediatric type 2 diabetes mellitus (T2DM) is crucial for improving long‐term outcomes. This study aimed to assess if the severity of T2DM at presentation, inferred by the location of treatment initiation (inpatient or outpatient), influences long‐term clinical outcomes.

**Methods:**

A retrospective chart review was conducted on 116 pediatric T2DM patients. Data on treatment initiation location, initial and subsequent glycated hemoglobin (HbA1c) levels, prescribed insulin, and body mass index were collected from electronic medical records.

**Results:**

Of the 116 patients, 69 were initially treated in an inpatient setting, and 47 received outpatient treatment. At treatment initiation, the inpatient group had significantly higher HbA1c levels compared to the outpatient group (*p* < .001), but 3 years after treatment initiation, no significant difference in HbA1c was observed between the two groups (*p* = .057). Prescribed insulin dosages were higher in the inpatient group at treatment initiation (*p* < .001) and remained higher after 3 years (*p* < 0.003) compared to the outpatient group.

**Conclusions:**

Pediatric patients initially treated in an inpatient setting had poorer glycemic control and higher prescribed insulin dosing at baseline. After 3 years, there was no significant difference in HbA1c levels, but patients treated as inpatients continued to have higher prescribed insulin. These findings suggest that the severity of diabetes at initial presentation may affect long‐term clinical outcomes in children with T2DM.

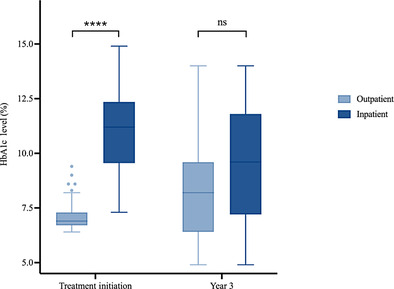

## Graphical abstract

Highlights

- This study assesses if the severity of type 2 diabetes in children at presentation, inferred by the location of treatment initiation (inpatient vs outpatient) influences long‐term clinical outcomes.
- Children with new‐onset type 2 diabetes treated in an inpatient setting presented with higher glycated hemoglobin (HbA1c) levels at initiation and were treated with higher doses of insulin.
- After 3 years, there was no significant difference in HbA1c levels between inpatient and outpatient groups.
- However, patients initially treated inpatient continued to have higher prescribed insulin dosages after 3 years of diagnosis.

## 1 INTRODUCTION

Type 2 diabetes mellitus (T2DM) is affecting an increasing number of pediatric patients even as rates of child and adolescent obesity have plateaued, with the burden falling predominantly on racial minorities and those of low socioeconomic status.^1^, ^2^ The pathophysiology underlying the development of pediatric T2DM is multifactorial. Genetic predisposition, intrauterine environment, excessive food consumption, weight gain, and increased sedentary behavior all contribute to the development of insulin resistance and associated metabolic derangements.^3^ In children and adolescents, high therapeutic failure rates, decreased response to insulin sensitizers, and a more rapid progression to both microvascular and macrovascular complications relative to adults suggest a more severe disease process.^3^, ^4^, ^5^ Given the increasing prevalence of pediatric T2DM and its associated complications, improved recognition, screening, and treatment strategies for children with T2DM are needed to reduce its impact.

Despite relatively varied treatment options for adult T2DM, pharmacologic therapies for pediatric T2DM remain limited, and strategies for optimal management are not well established. Unless contraindicated, metformin is recommended for all children with T2DM.^4^ However, the Treatment Options for Type 2 Diabetes in Adolescents and Youth (TODAY) study demonstrated that treatment failure (glycated hemoglobin [HbA1c] ≥ 8% over a period of 6 months) occurred in approximately half of patients.^5^ Insulin is another mainstay of therapy, but its use is complicated by regimen complexity and associated adverse effects of weight gain and hypoglycemia. Pediatric patients are diagnosed with T2DM at varying stages of the disease process and subsequently require differing levels of therapy. Given that the benefits of metformin and insulin are mixed, further evidence is needed to characterize how these treatments affect specific cohorts.

The triage and initial treatment strategy and setting of initial care for youth with T2DM are not well established or described. Patients admitted inpatient for initial treatment of T2DM are typically those that have a more severe presentation, presenting with higher HbA1c levels or in diabetic ketoacidosis, which is concerning for a more advanced disease stage. It is important to understand what factors led to a more severe presentation and what that presentation may mean for future prognosis. Studies comparing outpatient management to inpatient management of children with newly diagnosed type 1 diabetes have not shown significant differences in long‐term glycemic control.^6^ However, the disease course in type 1 diabetes and type 2 diabetes is different and thus the findings cannot be extrapolated.

This study was designed to assess if the severity of T2DM at treatment initiation is related to long‐term glycemic control and prescribed insulin in children. The primary objective was to understand the impact of acuity of initial T2DM at presentation on HbA1c levels and prescribed insulin over 3 years of follow‐up.

## 2 MATERIALS AND METHODS

### 2.1 Study design

We performed a retrospective chart review. Charts were initially screened by *International Classification of Diseases, Ninth Revision* (ICD9) or *Tenth Revision* (ICD10) codes for diabetes (ICD9 250.00, ICD10 E13.9, or E11.9) between January 1, 2021 and November 1, 2021. This query produced 513 patients. Patients were eligible to be included in the study if they met the following criteria: (a) aged 8–18 years who were treated for new‐onset T2DM between January 1, 2021 and November 1, 2021, (b) received their diabetes care from an endocrinologist within the Ann & Robert H. Lurie Children's Hospital of Chicago system, and (c) were followed for greater than 2.5 years with at least three visits occurring during the follow‐up period. The charts were then validated by the research team to confirm the clinical diagnosis of T2DM and to confirm that patients met all eligibility criteria. Those with other forms of diabetes mellitus such as type 1 diabetes, drug‐induced diabetes, neonatal diabetes or maturity onset diabetes of youth, or other chronic illnesses that could alter the disease process such as Down syndrome, cystic fibrosis, Prader Willi syndrome, Turner's syndrome, history of cancer, or history of transplant were excluded.

Ultimately, 116 patients were included for analysis. The patients identified by ICD codes that were eventually excluded were excluded for the following reasons: 36 diagnosed before the study period, 62 not followed for >2.5 years with minimum of three visits, 248 with other forms of diabetes diagnoses, 25 with other complicating medical diagnoses, and 26 with incomplete diagnostic information or not meeting age criteria.

This study was conducted in accordance with the Declaration of Helsinki and was approved by the Lurie Children's Hospital Institutional Review Board prior to any data collection.

Demographic, clinical, and laboratory data were obtained from the electronic medical record. Demographic data included patient age, sex, race, ethnicity, and insurance provider. Clinical data included encounter location, length of hospitalization, initial medical therapy, insulin dosing, height, weight, and body mass index (BMI). Laboratory measures included HbA1c, venous blood gas pH, bicarbonate (mEq/L), glucose (mg/dL), urine ketones, beta‐hydroxybutyrate (mmol/L), and diabetes‐associated autoantibodies.

The criteria used to define T2DM diagnosis in this studywere HbA1c greater than or equal to 6.5% (48 mmol/mol), OR oral glucose tolerance test results (fasting >126 mg/dL OR 2 h > than 200 mg/dL), OR a random blood glucose of >200 mg/dL with consistent symptomatology. An HbA1c of ≤6.5% was used to define optimal glycemic control based on International Society for Pediatric and Adolescent Diabetes guidelines.^7^ Durable glycemic control was defined by a HbA1c ≤ 8% as classified in the TODAY trial.^5^

### 2.2 Statistical analyses

We performed all statistical analyses using RStudio version 1.2.1335. Patient characteristics were summarized with frequencies and percentages for categorical variables, means ± SD for normally distributed continuous variables, and medians and interquartile ranges (IQR) for nonnormally distributed continuous variables. Normality testing was performed using Shapiro–Wilk test. To assess differences in demographic and clinical characteristics between the inpatient and outpatient groups, we used the two‐sample *t* test or the Mann–Whitney *U* test and the Pearson's chi‐square test for continuous measures and categorical characteristics, respectively. Univariate logistic regression analyses were used to assess odds of achieving durable and optimal glycemic control over the 3‐year follow‐up period in the inpatient group compared to the outpatient group. The Mann–Whitney *U* test was used to compare HbA1c and insulin dose at initial and final presentations between groups, respectively. The Friedman test was used to assess for differences in the HbA1c values within the groups over the four time points measured (baseline, year 1, year 2, and year 3), and post hoc tests using the pairwise Mann–Whitney *U* test with a Bonferroni correction were performed to explore which time points differ from one another.

## 3 RESULTS

The characteristics of the 116 patients are shown in Table 1. Out of a total of 116 patients included in the analysis, 69 patients were initially treated with T2DM in an inpatient setting and 47 patients were initially treated in an outpatient setting. There were no significant differences in demographic characteristics across the two groups. The median age (IQR) of the patients at treatment initiation was 13.1 (11.6–14.7) years and the median age at the final encounter was 16.4 (14.6–18.0) years. Patients were followed for a median time of 3.2 (3.0–3.3) years. Among the 116 patients, the majority were Hispanic (75%) and female (62%). A total of 10 patients presented at treatment initiation with diabetic ketoacidosis.

**TABLE 1:** Patient characteristics stratified by presentation setting.

	All patients, *N* = 116	Inpatient group, *N* = 69 (59.5%)	Outpatient group, *N* = 47 (40.5%)	*p* value^a^
	Demographics and characteristics			
Sex, *n* (%)				.364
Male	44 (37.9)	29 (42.0)	15 (32.0)	
Female	72 (62.1)	40 (58.0)	32 (68.0)	
Insurance, *n* (%)				.183
Medicaid	69 (59.5)	45 (65.2)	24 (51.0)	
Not Medicaid	47 (40.5)	24 (34.8)	23 (49.0)	
Race or ethnicity, *n* (%)				.766
Hispanic	87 (75.0)	49 (71.0)	38 (80.9)	
Non‐Hispanic Black	17 (14.7)	12 (17.4)	5 (10.6)	
Non‐Hispanic White	6 (5.2)	4 (5.8)	2 (4.3)	
Non‐Hispanic Asian	6 (5.2)	4 (5.8)	2 (4.3)	
Age at treatment initiation in years, median (IQR)	13.1 (11.6–14.7)	13.5 (11.4–14.7)	13.0 (11.7–14.6)	.761
Age at follow‐up in years, median (IQR)	16.4 (14.6–18.0)	16.49 (14.5–18.0)	16.01 (14.8–17.8)	.862
Years followed, median (IQR)	3.2 (3.0–3.3)	3.14 (3.0–3.3)	3.17 (3.0–3.3)	.355
BMI *Z*‐score at treatment initiation, median (IQR)	2.3 (2.1–2.6)	2.25 (2.0–2.5)	2.39 (2.2–2.6)	**.022**
DKA condition at treatment initiation, *n* (%)				**.005**
DKA	10 (8.6)	10 (14.5)	0 (0)	
Non‐DKA	106 (91.4)	59 (85.5)	47 (100)	
	Initial Treatments			
Metformin, *n* (%)				**<.001**
Metformin	45 (38.8)	12 (17.4)	33 (70.2)	
Metformin not prescribed	71 (61.2)	57 (82.6)	14 (29.8)	
Insulin prescribed^b^, *n* (%)				**<.001**
Insulin prescribed	70 (60.3)	65 (94.2)	5 (10.6)	
Insulin not prescribed	46 (39.7)	4 (5.8)	42 (89.4)	
GLP‐1 agonist, *n* (%)				
GLP‐1 agonist	0 (0)	0 (0)	0 (0)	1.000
GLP‐1 agonist not prescribed	116 (100)	69 (100)	47 (100)	
	Treatments at year 3^c^			
Metformin, *n* (%)				.403
Metformin	75 (64.7)	42 (60.9)	33 (70.2)	
Metformin not prescribed	41 (35.3)	27 (39.1)	14 (29.8)	
Insulin prescribed, *n* (%)				**.028**
Insulin prescribed	81 (69.8)	54 (78.3)	27 (57.4)	
Insulin not prescribed	35 (30.2)	15 (21.7)	20 (42.6)	
GLP‐1 agonist, *n* (%)				0.647
GLP‐1 agonist	5 (4.30)	4 (5.8)	1 (2.1)	
GLP‐1 agonist not prescribed	111 (95.7)	65 (94.2)	46 (97.9)	

*Note*: Data are expressed as *n* (%) or median (IQR). A *p* < 0.05 was considered statistically significant (Bold).

Abbreviations: BMI, body mass index; DKA, diabetic ketoacidosis; GLP‐1, glucagon‐like peptide‐1; IQR, interquartile range; SGLT2, sodium/glucose cotransporter 2.

^a^ The *p* value indicates the level of significance of the differences observed between the inpatient and outpatient groups.

^b^ Insulin prescribed represents that the patient used any type of insulin, including basal, bolus or mixed insulin.

^c^ Two patients were prescribed SGLT2 inhibitors at follow‐up. It is unclear whether prescribed for diabetes or other indications. SGLT2 inhibitors were not Food and Drug Administration approved for diabetes treatment in pediatrics at the time of the study.

At the end of year 1, 85.1% of the patients initially treated in an outpatient setting achieved durable glycemic control whereas 55.1% of the patients initially treated in an inpatient setting achieved durable glycemic control (Table 2). The odds of achieving durable glycemic control were decreased in patients initially treated in an inpatient setting compared to those treated in an outpatient setting (odds ratio [OR] = 0.21, 95% confidence interval [CI]: 0.08–0.52, *p* = 0.001).

**TABLE 2:** Differences in glycemic control at the end of year 1 between patients initially treated in an inpatient setting and patients initially treated in an outpatient setting.

	All patients, *N* = 116	Inpatient group, *N* = 69 (59.5%)	Outpatient group, *N* = 47 (40.5%)	OR (95% CI)	*p* value
Durable glycemic control (HbA1c ≤ 8%), *n* (%)				0.21 (0.08, 0.52)	**.001**
Yes	78 (67.2%)	38 (55.1%)	40 (85.1%)		
No	38 (32.8%)	31 (44.9%)	7 (14.9%)		
Optimal glycemic control (HbA1c ≤ 6.5%), *n* (%)				0.37 (0.17, 0.79)	**.011**
Yes	50 (43.1%)	23 (33.3%)	27 (57.4%)		
No	66 (56.9%)	46 (66.7%)	20 (42.6%)		

*Note*: A *p* < 0.05 was considered statistically significant (Bold).

Abbreviations: CI, confidence interval; HbA1c, glycated hemoglobin; OR, odds ratio.

Optimal glycemic control was achieved by 57.4% of patients initially treated in an outpatient setting and 33.3% of patients initially treated in an inpatient setting at the end of year 1. Patients who were initially treated in an inpatient setting had decreased odds of achieving optimal glycemic control compared to those treated as an outpatient (OR = 0.37, 95% CI: 0.17–0.79, *p* = 0.011).

After 3 years of follow‐up, 50% of patients initially treated in an outpatient setting achieved durable glycemic control whereas 31.1% of patients initially treated in an inpatient setting achieved durable glycemic control (Table 3). Our analysis revealed that there was a significant difference in the odds of achieving durable glycemic control at year 3 between the inpatient and outpatient groups (OR = 0.46, 95% CI: 0.21–0.99, *p* = 0.047), with patients who were initially treated in an inpatient setting having decreased odds of achieving durable glycemic control.

**TABLE 3:** Differences in glycemic control at 3 years of follow‐up between patients who were initially treated in an inpatient setting and patients initially treated in an outpatient setting.

	All patients, *N* = 116	Inpatient group, *N* = 69 (59.5%)	Outpatient group, *N* = 47 (40.5%)	OR (95% CI)	*p* value
Durable glycemic control (HbA1c ≤ 8%), *n* (%)				0.46 (0.21, 0.99)	**.047**
Yes	44 (38.9%)	21 (31.1%)	23 (50%)		
No	69 (61.1%)	46 (68.7%)	23 (50%)		
Optimal glycemic control (HbA1c ≤ 6.5%), *n* (%)				0.66 (0.28, 1.55)	0.34
Yes	29 (25.7%)	15 (22.3%)	14 (30.4%)		
No	84 (74.3%)	52 (77.6%)	32 (69.6%)		

*Note*: A *p* < 0.05 was considered statistically significant (Bold).

Abbreviations: CI, confidence interval; HbA1c, glycated hemoglobin; OR, odds ratio.

With regard to optimal glycemic control at 3 years of follow‐up, 30.4% of the patients initially treated in an outpatient setting achieved it whereas 22.3% of the patients initially treated in an inpatient setting achieved it. No significant difference was found in the odds of achieving optimal glycemic control between the outpatient and inpatient groups at year 3 (OR = 0.66, 95% CI: 0.28–0.99, *p* = 0.34).

**FIGURE 1:**
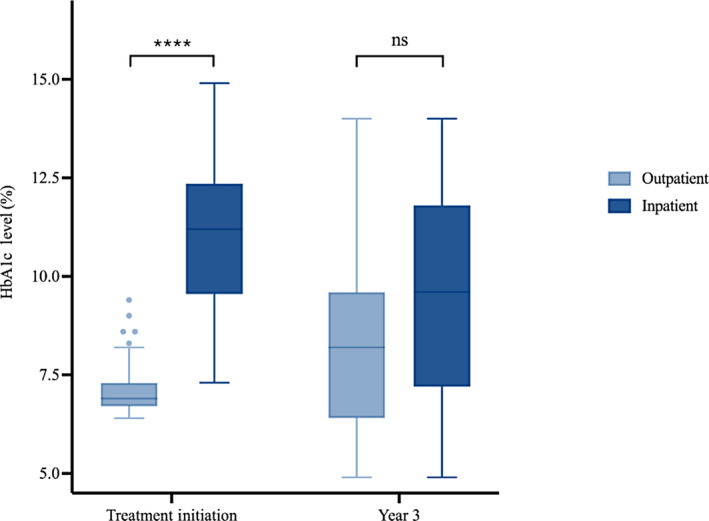
Comparison between inpatient and outpatient groups regarding HbA1c at treatment initiation and year 3, respectively (*: *p* < .05; **: *p* < .01; ***: *p* < .001; ****: *p* < 0.0001). HbA1c, glycated hemoglobin; ns, not statistically significant).

Diabetes presentation was more severe at treatment initiation in the inpatient group compared to the outpatient group (Figure 1). At treatment initiation, HbA1c levels in the inpatient group (median = 11.2%, IQR: 9.60%–12.30%) were higher than in the outpatient group (median = 6.9%, IQR: 6.70%–7.30%; *p* < 0.001). The difference in disease severity that existed at baseline, however, did not persist after 3 years of follow‐up. Three years after treatment initiation, the difference in HbA1c levels between the inpatient group (median = 9.60%, IQR: 7.30%–11.70%) and outpatient group (median = 8.2%, IQR: 6.43%–9.50%) was no longer significant (*p* = 0.057).

**FIGURE 2:**
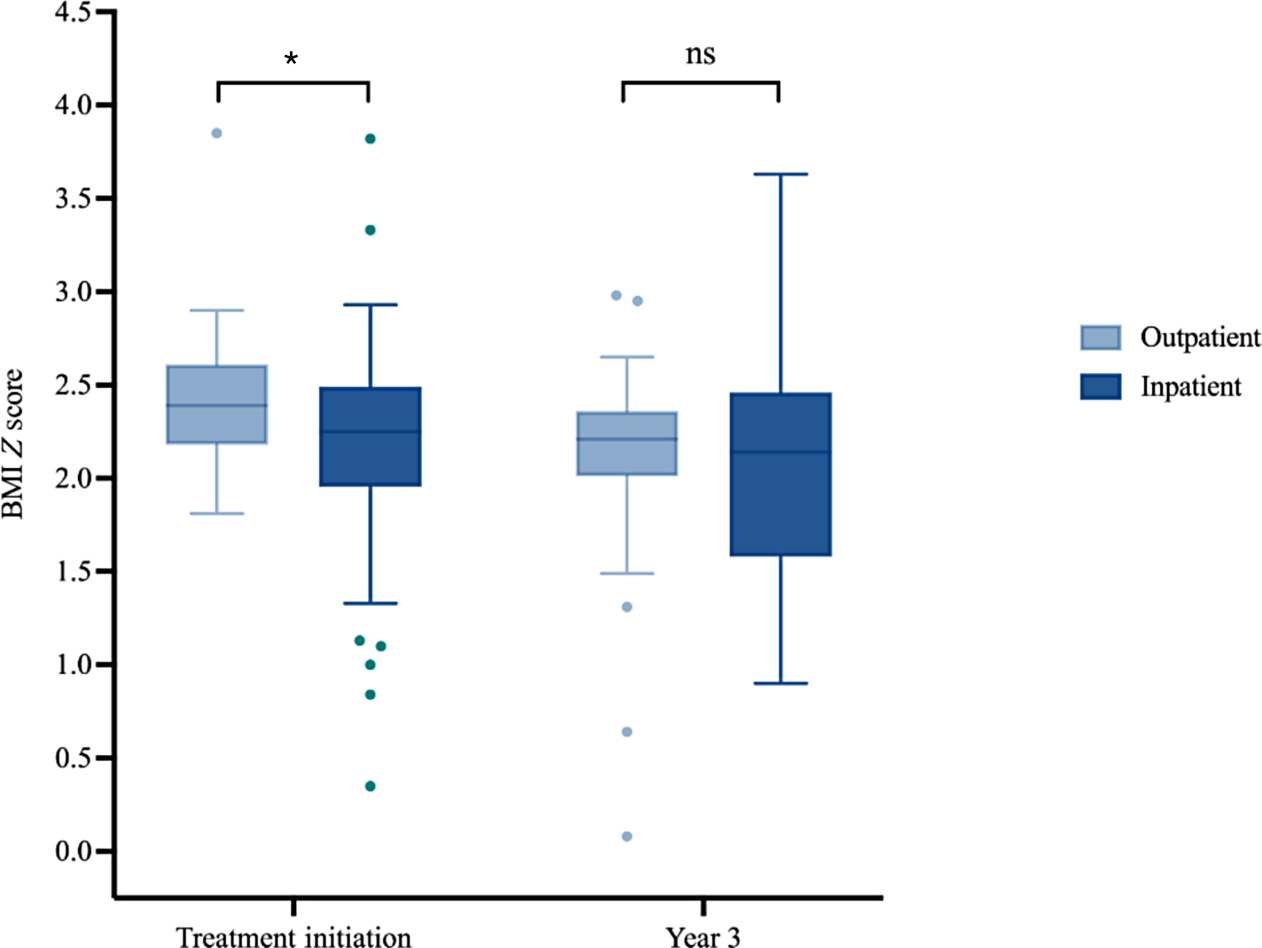
Comparison between inpatient and outpatient groups regarding BMI *Z*‐score at treatment initiation and year 3, respectively (*: *p* < .05; **: *p* < .01; ***: *p* < .001; ****: *p* < .0001). BMI, body mass index; HbA1c, glycated hemoglobin; ns, not statistically significant).

The patients who were first treated inpatient also presented with lower BMI (Figure 2). The BMI *Z*‐scores at initial presentation were lower in the inpatient group (median = 2.25, IQR: 2.01–2.47) than the outpatient group (median = 2.39, IQR: 2.20–2.60; *p* = 0.022). At 3 years of follow‐up, however, the difference in BMI *Z*‐score between the inpatient and outpatient groups was also no longer significantly different (*p* = .27).

**FIGURE 3:**
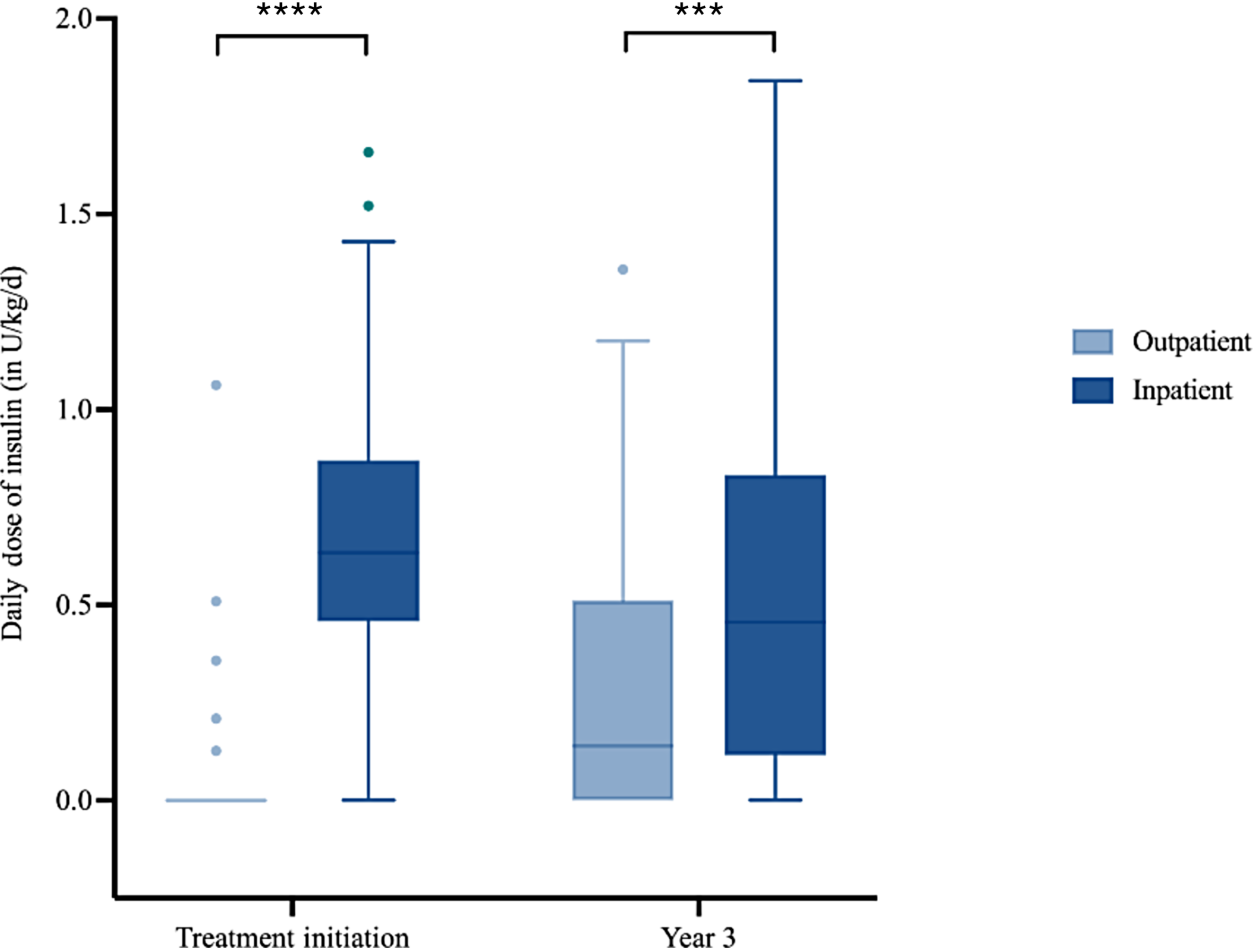
Comparison between inpatient and outpatient groups regarding BMI *Z*‐score at treatment initiation and year 3, respectively (*: *p* < .05; **: *p* < .01; ***: *p* < .001; ****: *p* < .0001). BMI, body mass index; ns, not statistically significant.

The more advanced nature of the disease process in the inpatient group also required more intensive therapy (Figure 3). At treatment initiation the average total daily dose of insulin per kg of body weight in the inpatient group (median = 0.63 U/kg/d, IQR: 0.49 U/kg/d – 0.84 U/kg/d) was higher than the outpatient group (median = 0 U/kg/d, IQR: 0 U/kg/d – 0 U/kg/d; *p* < 0.001). Unlike the differences in glycemic control and BMI *Z*‐score, the increased prescribed insulin dosing within the inpatient group persisted over the follow‐up period. At 3 years of follow‐up the average total daily dose of insulin remained higher in the inpatient group (median = 0.011 U/kg/d, IQR: 0.008 U/kg/d – 0.013 U/kg/d) compared to the outpatient (median = 0.008 U/kg/d, IQR: 0 U/kg/d – 0.011 U/kg/d; *p* = .0025) group.

**FIGURE 4:**
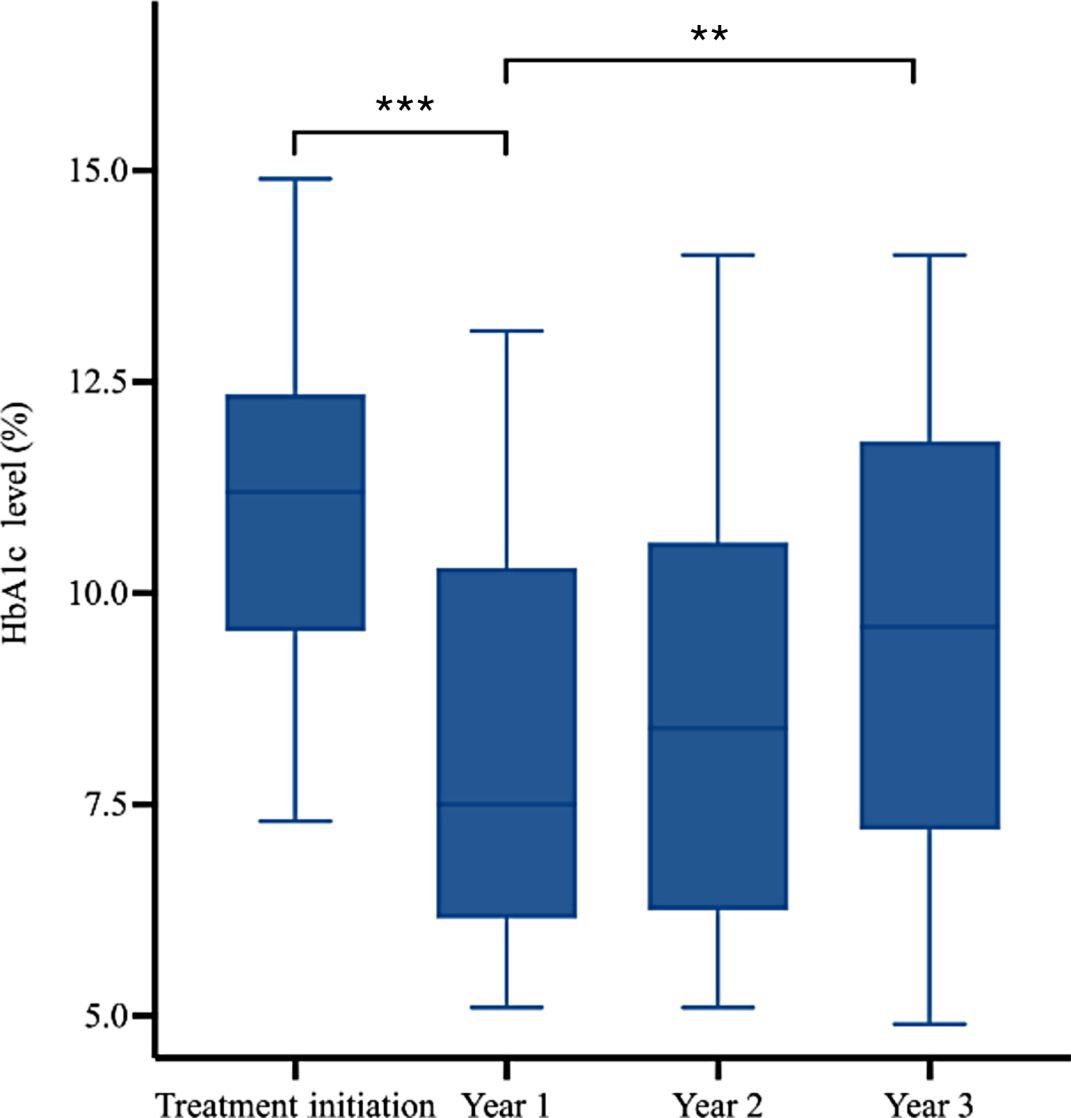
HbA1c levels over 3 years of follow‐up for patients initially treated in inpatient settings (*: *p* < .05; **: *p* < .01; ***: *p* < .001; ****: *p* < .0001. HbA1c, glycated hemoglobin; ns, not statistically significant.

**FIGURE 5:**
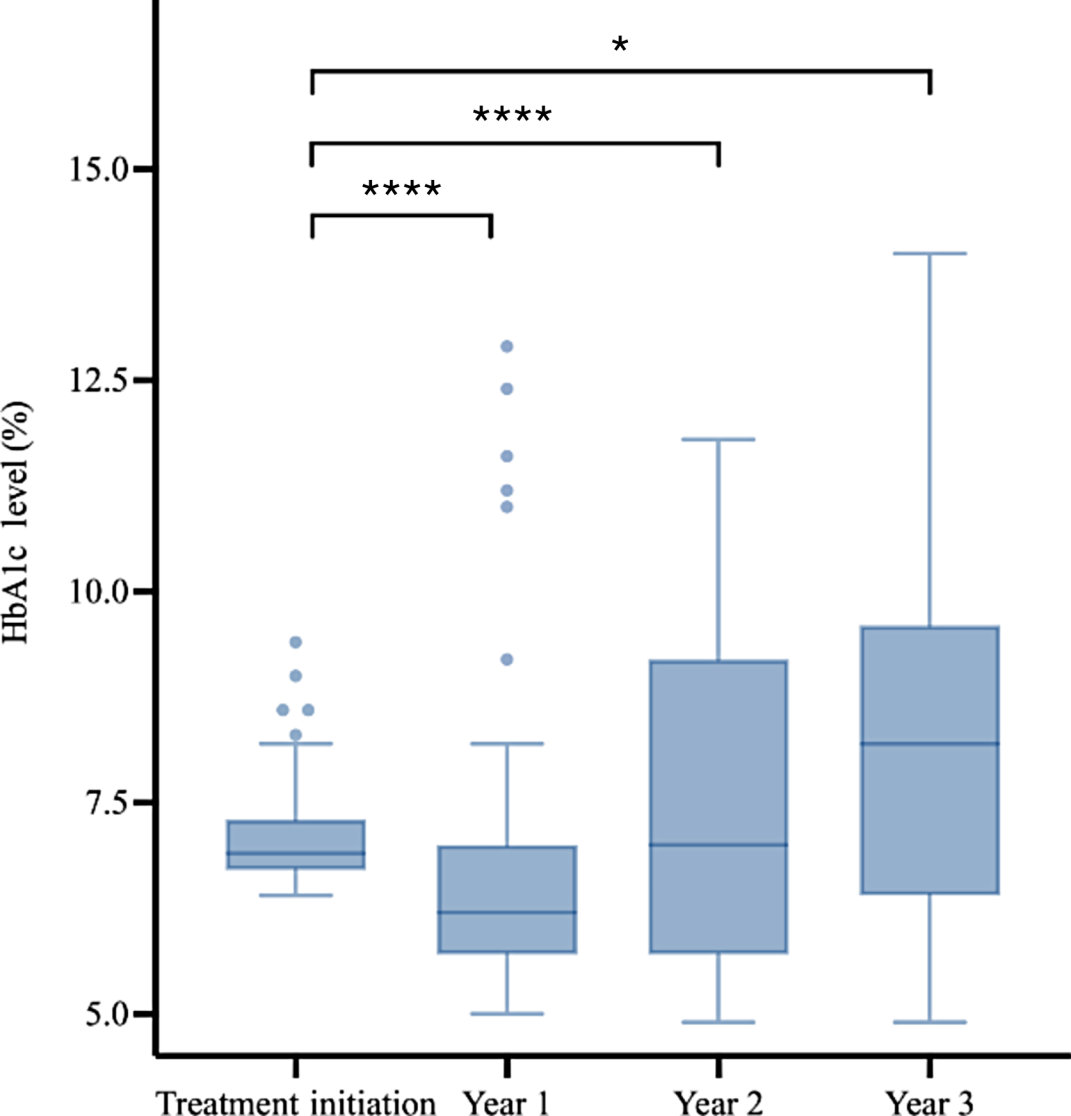
HbA1c levels over 3 years of follow‐up for patients initially treated in outpatient settings (*: *p* < .05; **: *p* < .01; ***: *p* < .001; ****: *p* < .0001.) HbA1c, glycated hemoglobin; ns: not statistically significant.

The trend in glycemic control of patients included in the study was marked by improvements in HbA1c levels after 1 year, followed by a gradual increase in HbA1c over the next 2 years (Figures 4 and 5). The Friedman and post hoc tests demonstrated significant differences among the HbA1c levels over the four time points for both the inpatient and outpatient groups.

**FIGURE 6:**
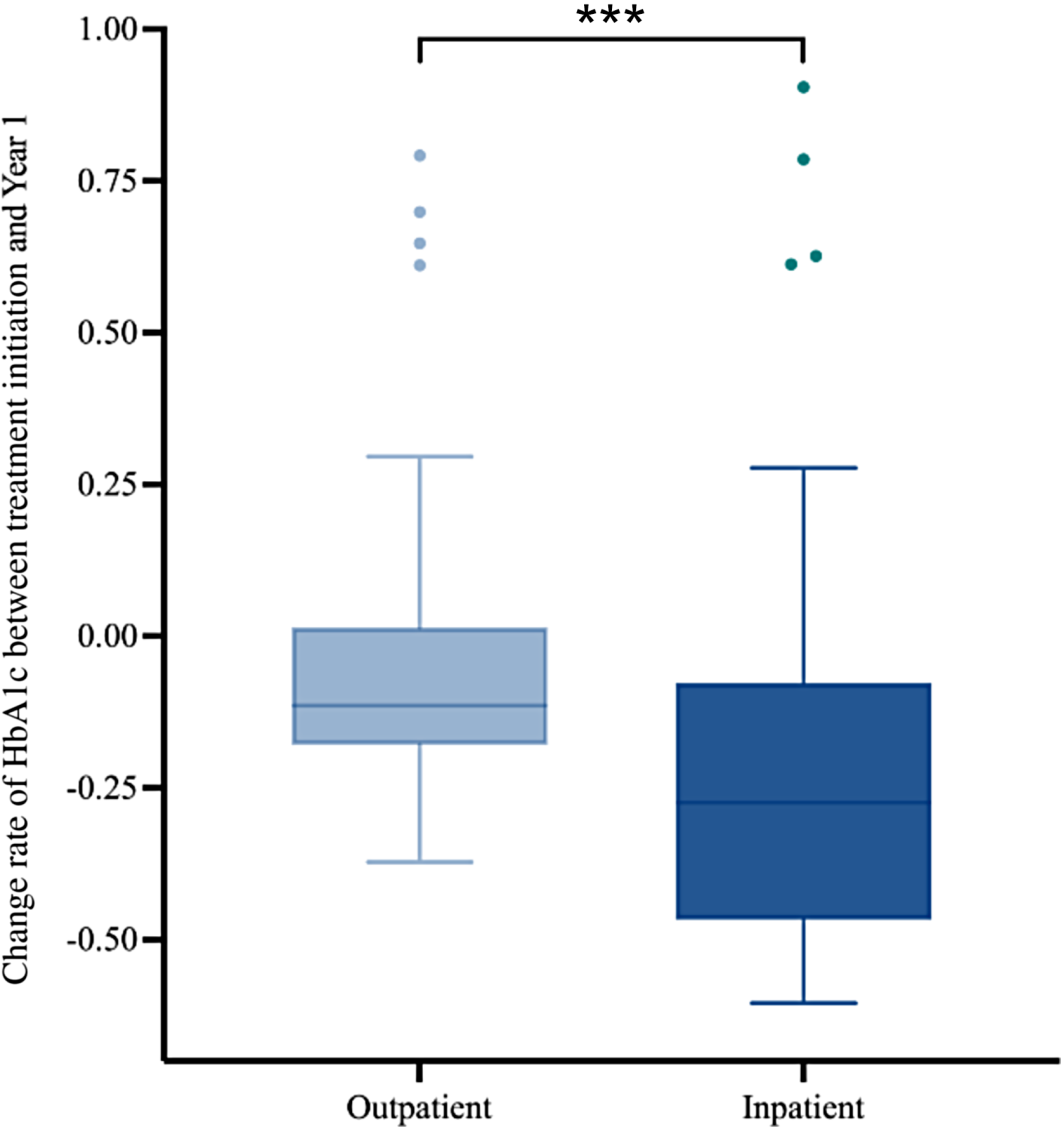
Change of HbA1c in percentage between the treatment initiation and the first year of follow‐up (*: *p* < .05; **: *p* < .01; ***: *p* < .001; ****: *p* < .0001). HbA1c, glycated hemoglobin; ns: not statistically significant.

Notably, both the inpatient and outpatient groups demonstrated significant decreases in HbA1c levels in the first year following treatment. Compared with the HbA1c level at treatment initiation, the HbA1c level decreased at year 1 for both inpatient and outpatient groups, with the HbA1c level in the inpatient group (median = −27%, IQR: −46% to −8%) decreasing at a higher rate than in the outpatient group (median = −11%, IQR: −18%–1%; *p* < .001) (Figure 6).

## 4 DISCUSSION

This study describes how the severity of initial presentation was related to glycemic control and prescribed insulin of children with type 2 diabetes treated at a single institution over 3 years. In this study, the patients presenting inpatient for treatment had higher HbA1c levels (median = 11.2%) in comparison to those initially seen as outpatients who presented with less severe dysglycemia (median = 6.9%). Additionally, the inpatient group initially presented with significantly lower BMI *Z*‐scores compared to the outpatient group likely secondary to the inability to utilize glucose resulting in weight loss and in some cases diabetic ketoacidosis in the inpatient group. These data, taken together, are evidence of a more severe disease state in the inpatient group. As studies in adults and in the TODAY cohort have both demonstrated, β‐cell function is a strong determinant of HbA1c.^8^, ^9^ Children initially treated in an inpatient setting for type 2 diabetes are likely to be identified at a state of greater β‐cell function compromise, with impacts on the long‐term metabolic outcomes and treatment strategies.

Preservation of β‐cell function has been described with early initiation of insulin therapy.^10^, ^11^ Shorter exposure to periods of hyperglycemia and associated glucotoxicity are associated with greater likelihood of restoration of β‐cell function.^8^ In our study population, patients treated in the inpatient setting were more often started on insulin therapy and treated more intensively. In the inpatient group, the differences between the HbA1c at first treatment and the HbA1c at years 1, 2, and 3 were all found to be significant, indicating that following treatment initiation, the level of glycemic control improved at year 1 and remained significantly lower at years 2 and 3. When we compared the time at treatment initiation and at 3 years of follow‐up, they were prescribed more insulin compared to patients treated in the outpatient setting. The data also demonstrate that the HbA1c level decreased at year 1 for both inpatient and outpatient groups but decreased at a higher rate in the inpatient group, likely due to the more intensive management with insulin therapy in the inpatient group. Despite more intensive therapy at the time of treatment initiation, patients initially treated in an inpatient setting were likely exposed to longer periods of glucotoxicity, with significant and irreversible baseline deterioration in β‐cell function at the time of treatment.

In the outpatient group, the level of glycemic control following treatment initiation also improved in the first year, but at years 2 and 3, the level of glycemic control was no longer significantly different from year 1, suggesting that the initial improvements in glycemic control observed in the outpatient group were not sustained over the follow‐up period. In the outpatient group, the preference for initial treatment with metformin, which has demonstrated high treatment failure rates,^5^ may have prolonged exposure to glucotoxicity, such that when insulin was initiated, β‐cell function was once again significantly impaired. These results are in line with data from previous studies demonstrating that adolescents with type 2 diabetes who do not attain a nondiabetes range HbA1c after a few months on metformin are at increased risk for losing glycemic control.^12^ However, it is difficult to assess whether this was related to initial treatment choice or subsequent treatment decisions as those treated initially as an outpatient were likely to have lower amounts of prescribed insulin both at treatment initiation and at follow‐up despite having HgbA1C values that were not significantly different at 3‐year follow‐up than those initially treated in the inpatient setting. Therefore, it may be that providers experienced “treatment inertia” and were less likely to discontinue insulin for patients in whom insulin was already prescribed and less likely to initiate insulin for those who were not prescribed insulin at treatment initiation. Regardless, the significant proportion of patients experiencing treatment failure in both groups may be a result of delayed treatment escalation either at treatment initiation or at follow‐up or a failure to titrate insulin, particularly basal insulin, to adequate dosing levels. Additionally, it may be related to relatively low levels of metformin use. At treatment initiation, 17% of those treated as inpatients and 70% of those treated as outpatients received metformin. At 3 years of follow‐up this rate was increased in the inpatient group but stable in the outpatient group, with only 60% of those treated as an inpatient and 70% of those treated as an outpatient prescribed metformin. The reasons that metformin was not prescribed are beyond the scope of this article but likely include patient preference and intolerance of medication.

Overall, very few patients achieved optimal glycemic control of a HbA1c ≤ 6.5% after 3 years. The first year of follow‐up demonstrated notable improvements in glycemic control in both the inpatient and outpatient groups. The improvement in glycemic control was sustained in the inpatient group over the subsequent 2 years, but the outpatient group showed no significant improvement in glycemic control over the follow‐up period. These findings are consistent with data previously published in the TODAY study. In the TODAY study, among those receiving metformin monotherapy treatment, the treatment failure rate was approximately 50% after an average follow‐up period of 3.86 years.^5^ In our study, the proportion of patients with HbA1c levels >8% at 3 years was 61.1%, with a failure rate of 50% in the outpatient group and 68.7% in the inpatient group.^5^ These data suggest that the current treatment options for pediatric T2DM are unable to halt the progression of T2DM in most youth. The findings also suggest that short‐term improvements in glycemic control in the first year do not prevent β‐cell function decline in the long‐term regardless of initial disease severity.

Unfortunately, in this study medication regimens were not standardized, and medication compliance was unavailable. Literature has proposed some of these as possible explanations for these trends^13^, ^14^; however, it is likely that baseline β‐cell function, not medication nonadherence, is the major predictor of long‐term glycemic outcomes.^5^ Previous studies have demonstrated rapid deterioration in β‐cell function, as well as an inability to stop or reverse the progressive decline in youth diagnosed with T2DM. The TODAY study data, for example, showed a 20%–35% decline in β‐cell function per year, which contrasts with a decline of 7%–11% per year in adult studies such as A Diabetes Outcome Progression Trial (ADOPT).^15^ A question for further study is whether earlier recognition of treatment failure in the outpatient groups could have led to better glycemic outcomes and less treatment failure at 3‐year follow up. Furthermore, glucagon‐like peptide‐1 (GLP1) treatment was not initiated as frequently as current practice and it would be interesting to reassess HbA1c with GLP1 use in a few years.

## 5 STRENGTHS AND LIMITATIONS

This study is one of the first outcomes reports to look at how presentation location affects long‐term outcomes in pediatric T2DM. One strength is the fact that the data reflect glycemic trends in youth with type 2 diabetes being treated in a real‐world setting. Adjustments to treatment regimens were made at individual encounters throughout the course of the follow‐up period by endocrinologists seeking to optimize glycemic control, and the data reflect these changes. The care delivered to the patients occurred at a single institution and is therefore reflective of consistent, shared approaches to management, despite different endocrinologists providing the care. All patients received similar nutritional counseling and lifestyle management. This study population is also significantly composed of Hispanic youth, a population known to disproportionately be affected by pediatric type 2 diabetes.

The limitations of the study include a relatively small sample size and retrospective design. Given the retrospective design, patients with incomplete data were excluded. A significant concern is selection bias resulting from the exclusion of patients who did not have 3 years of follow‐up (62 of the original 513 patients identified by ICD code). It is possible that patients with more severe disease returned to receive care compared to those with mild disease processes. Data from this study come from a large pediatric center serving a vast area and patients likely present with more severe disease processes. As with all retrospective studies, associations measured in this study are subject to confounding by risk factors not measured. For example, there was a lack of collection of additional social determinants of health (parental employment status, education level, food insecurity) which are known to affect outcomes in pediatric T2DM.

## 6 CONCLUSIONS

Most children in our study required continued treatment at 3 years of follow‐up. The pediatric T2DM patients in our cohort did achieve reductions in the HbA1c level in the inpatient group over 3 years, but patients in the outpatient group did not. The results of our study suggest that pediatric patients initially treated in the inpatient setting have poorer glycemic control at baseline when compared to patients treated outpatient. Although higher insulin use in the inpatient group persisted after 3 years of follow‐up, the difference in glycemic control did not, suggesting that more insulin use in the inpatient group, either at treatment initiation or follow‐up, led to more significant reductions in HbA1C and similar levels of disease control at follow‐up. Obtaining glycemic control is an important part of the management of pediatric T2DM, and these findings suggest that initial presentation may be an important factor when predicting long‐term clinical outcomes in children with T2DM.

## AUTHOR CONTRIBUTIONS

All listed authors contributed significantly to this work and are in agreement with the content of the manuscript. Adesh Ranganna and Monica E. Bianco conceived the study concept and design. All authors participated in validating the data and took part in interpretation of the results. Adesh Ranganna and Monica E. Bianco were primarily responsible for preparing the manuscript. Wenya Chen was primarily responsible for the statistical modeling. All authors participated in manuscript editing and revisions of the manuscript.

## FUNDING INFORMATION

MB's work was partially supported by an adminstrative supplement (3 R01DK118403‐02S1). Research involved the use of REDCap at Northwestern Univeristy Clinical translational Sciences (NUCATs) Institute funded by a Clinical and Translational Science Award (CTSA) grant from the National Institutes of Health (NIH) UL1TR00142. Research was also supported by a voucher from the Stanley Manne Children's Research Institute at Ann & Robert H. Lurie Children's Hospital of Chicago.

## CONFLICT OF INTEREST STATEMENT

The authors declare that there is no conflict of interest regarding the publication of this paper.

## Data Availability

The data used to support the findings of this study are restricted by Ann & Robert H. Lurie Children's Hospital of Chicago Institutional Review Board in order to protect patient privacy. Data are available from Monica E. Bianco, MD, for researchers who meet the criteria for access to confidential data.
